# Non-Homogeneous Tumor Growth and Its Implications for Radiotherapy: A Phenomenological Approach

**DOI:** 10.3390/jpm11060527

**Published:** 2021-06-09

**Authors:** Paolo Castorina, Luigi Castorina, Gianluca Ferini

**Affiliations:** 1 Istituto Nazionale Fisica Nucleare, 95100 Catania, Italy; 2 Istituto Oncologico del Mediterraneo, 95029 Viagrande, Italy; 3 Faculty of Mathematics and Physics, Charles University, 18000 Prague, Czech Republic; 4 REM, 95029 Viagrande, Italy; luigi.castorina@grupposamed.com (L.C.); gianluca.ferini@grupposamed.com (G.F.)

**Keywords:** tumor instability, radiotherapy, dose-boost

## Abstract

Tumor regrowth and heterogeneity are important clinical parameters during radiotherapy, and the probability of treatment benefit critically depends on the tumor progression pattern in the interval between the fractional irradiation treatments. We propose an analytic, easy-to-use method to take into account clonal subpopulations with different specific growth rates and radiation resistances. The different strain regrowth effects, as described by Gompertz law, require a dose-boost to reproduce the survival probability of the corresponding homogeneous system and for uniform irradiation. However, the estimate of the survival fraction for a tumor with a hypoxic subpopulation is more reliable when there is a slow specific regrowth rate and when the dependence on the oxygen enhancement ratio of radiotherapy is consistently taken into account. The approach is discussed for non-linear two-population dynamics for breast cancer and can be easily generalized to a larger number of components and different tumor phenotypes.

## 1. Introduction

A quantitative understanding of tumor growth is crucial for the clinical management of disease, and tumor size is a main determinant of clinical severity and an important factor, among other criteria [1], to assess the staging criteria before and during radiotherapy (RT). Tumor regrowth during radiotherapy is, therefore, an important clinical parameter [2] and, in particular, the dose-response relationship and, thus, the probability of treatment benefit critically depend on the tumor heterogeneity and the regrowth pattern in the interval between the fractional irradiation treatments.

To clearly evaluate the clinical results, the tumor cell survival fraction, *S*, after *n* irradiations at dose per fraction *d*, in the overall treatment time *t*, is usually written as

(1) $$-ln\left(S\right)=n\left(\alpha d+\beta d^{2}\right)-\gamma t$$

and depends on the tumor radiosensitivity, expressed by the parameters α and β, according to the linear-quadratic model, and on the regrowth parameter $\gamma =ln2/\tau_{eff}$, where $\tau_{eff}$ is the the average clonogenic doubling time [3]. The above equation is, up to now, the usual basis for RT scheduling, and would predict the probability *P* of tumor control, defined as P=exp[−cS], with *c* as the clonogen number.

The underlying assumption of the general approach in Equation (1) is the uniform response to the therapy and a common specific regrowth rate of the whole set of cancer cells. Concerning the regrowth during radiotherapy, one has that: (a) the untreated tumor growth has been usually described by means of the Gompertz law (GL) [4,5,6,7,8], a non linear growth pattern previously proposed in actuarial mathematics [9]; (b) in a transplantable rat tumor, it was shown that control and regrowth curves after radiotherapy could be fitted by the same Gompertzian law, provided adjustments for the initial lag and the estimated number of clonogens immediately after irradiation were performed [10]; (c) Gompertzian growh has been assumed to describe human tumor repopulation during fractional radiotherapy by Hansen et al. [11] and by O’Donougue [12].

Regarding the non-homogeneus response, an insufficient oxygen supply is at the root of the radioresistance phenomenon. Indeed, within bulky tumors, hypoxic and well-oxygenated clonal strains coexist, the first generally needing an escalated dose to achieve the same survival fraction with respect to the second. Alternatively, it would be mandatory to irradiate the tumor with the same high dose, but such an approach could be detrimental for nearby healthy tissues. Hence, the need to diversify the radiation dose within tumor volume on the basis of its oxygenation landscape: to boost hypoxic areas while limiting unnecessary high radiation dose to well-oxygenated subvolumes.

For this purpose, various instrumental options are being studied to identify hypoxic areas within tumors, thus, introducing the Oxygen Guided Radiation Therapy (OGRT) era [13]. Electron Paramagnetic Resonance is still not clinically available; however, its therapeutic value has been proven for fibrosarcoma in mice and in preliminary human experiences [14,15]. Indeed, Electron Paramagnetic Resonance images may direct the location of radiation tumor boosts to enhance tumor cures [16]. On the other hand, some PET imaging has already been verified to be effective in guiding oxygen-based radiotherapy [17,18,19] and to transform the radiotracer uptake into oxygen partial pressure maps, determining the volume of the hypoxic target (HTV).

In such a context, mathematical models can assist a radiation oncologist in dose prescription, for example by evaluating the integrated boost directed to hypoxic subvolumes [20]. In this article, we propose an analytic method, which considers cancer’s inherent clonal diversity both in non-homogenous specific regrowth rates and in the corresponding radioresistance to estimate the dose-boost of some subpopulations to maintain the same tumor control of homogeneous treatments.

Being aware of the constitutive heterogeneity within a tumoral tissue, we discuss a mathematical model of cell behavior affected by ionizing radiations and based on only two cell populations with opposite patterns of oxygenation: well-oxygenated versus hypoxic ones. Indeed, the homogeneous case is taken into account only to show the difference when the non-homogeneity is present. Moreover, the proposed algorithm can be easily generalized to a larger number of strains, corresponding to different levels of non-homogeneous tumor cell behavior.

## 2. Materials and Methods

The non-homogeneous tumor structure and its role in radiotherapy effects is quantitatively discussed in this section. General macroscopic growth laws for a cell population N(t) are solutions of the differential equation ( for a classification see [21])

(2) $$\frac{1}{N(t)}\frac{dN(t)}{dt}=f\left[N\left(t\right)\right]$$

where f(N) is the specific growth rate, and its *N* dependence describes the feedback effects during the time progression. If f(N)= constant, the growth follows an exponential pattern, with no limit for t→∞. On the other hand, a saturation is obtained by the Gompertz equation, i.e.,

(3) $$\frac{1}{N(t)}\frac{dN(t)}{dt}=\alpha_{g}-k_{g}\;\ln\frac{N(t)}{N_{0}}\;\mathrm{Gompertz}\;,$$

where $\alpha_{g}$ and $k_{g}$ are constants and $N_{0}$ is the initial value. By defining

(4) $$\alpha_{g}+k_{g}\ln N_{0}=k_{g}\ln N_{\infty },$$

one obtains

(5) $$\frac{1}{N(t)}\frac{dN(t)}{dt}=-k_{g}\;\ln\frac{N(t)}{N_{\infty }},$$

where $N_{\infty }$ is the carrying capacity, i.e., the steady state is reached for dN/dt=0, when *N* is equal to $N_{\infty }$.

The GL is the solution of the previous equation, i.e.,

(6) $$N\left(t\right)=N_{0}e^{ln\left(N_{\infty }/N_{0}\right)\left[1-exp\left(-k_{g}t\right)\right]}.$$

The evaluation of GL regrowth effects during radiotherapy for a homeogeneous tumor, considering the linear-quadratic model, is reported in Appendix A (where we also depict the growth pattern without radiotherapy). Immediately after *n* doses with the time interval Δτ, the depletion of the cell number is

(7) $$N\left(n\right)=N_{0}e^{ln\left(N_{\infty }/N_{0}\right)I\left(n\right)-\left(\alpha d+\beta d^{2}\right)L\left(n\right)}$$

where

(8) $$I\left(n\right)=1-e^{-\left(n-1\right)k_{g}\Delta t}$$

and

(9) $$L\left(n\right)=\frac{1-e^{-nk_{g}\Delta t}}{1-e^{-k_{g}\Delta t}}.$$

Equations (7)–(9) apply to a cell population with a unique specific regrowth rate. However, the tumor mass can be produced by strains with different clonal behaviors, and therefore the previous analysis has to be generalized.

For two-population ($N^{\left(1\right)}\left(t\right),N^{\left(2\right)}\left(t\right)$) dynamics, with $N\left(t\right)=N^{\left(1\right)}\left(t\right)+N^{\left(2\right)}\left(t\right)$, where each population evolves according the GL, with the parameters $k_{g}^{\left(1\right)},k_{g}^{2)},N_{\infty }^{\left(1\right)},and\;N_{\infty }^{\left(2\right)}$, the radiotherapy effects after *n* treatments depend on the balance between the two sets of cells. Let us define the fraction of initial population 1 as $N_{0}^{\left(1\right)}=\chi N_{0}$ (therefore $N_{0}^{\left(2\right)}=\left(1-\chi \right)N_{0}$) and its fraction of carrying capacity as $N_{\infty }^{\left(1\right)}=\rho N_{\infty }$ ($N_{\infty }^{\left(2\right)}=\left(1-\rho \right)N_{\infty }))$. Moreover, $k_{g}^{\left(1\right)}\neq k_{g}^{\left(2\right)}$.

After each dose, the two populations grow according to different patterns and, before the next dose, they contribute with distinct weights to the total number of cells. By iteraction, the final result for population 1 is (see Appendix A)

(10) $$N{\left(n\right)}^{\left(1\right)}=N_{0}^{\left(1\right)}e^{ln\left(\rho N_{\infty }/\chi N_{0}\right)I_{1}\left(n\right)-\left(\alpha_{1}d_{1}+\beta_{1}d_{1}^{2}\right)L_{1}\left(n\right)}$$

where

(11) $$I_{1}\left(n\right)=1-e^{-\left(n-1\right)k_{g}^{\left(1\right)}\Delta t}$$

and

(12) $$L_{1}\left(n\right)=\frac{1-e^{-nk_{g}^{\left(1\right)}\Delta t}}{1-e^{-k_{g}^{\left(1\right)}\Delta t}}.$$

Analogous formulas for the second cell group are obtained by the substitutions $\rho \to \left(1-\rho \right),\chi \to \left(1-\chi \right),k_{g}^{\left(1\right)}\to k_{g}^{2)},\alpha_{1}\to \alpha_{2},\beta_{1}\to \beta_{2},d_{1}\to d_{2}$. Notice that $\alpha_{1}\neq \alpha_{2}$ and $\beta_{1}\neq \beta_{2}$ describe different radiotherapy resistances of the two subpopulations, and $d_{1}\neq d_{2}$ is the dose-boost of the components.

Finally, $N{\left(n\right)}^{\left(1\right)}+N{\left(n\right)}^{\left(2\right)}=N\left(n\right)$ gives the analytic expression that is applied in the next section.

## 3. Results

Let us first consider the case of homogeneous radioresistance and different specific regrowth rates of the subpopulations. Their combined effect for hypoxic strain will be considered later on.

### 3.1. Uniform Radioresistance and Different Specific Growth Rates

**Figure 1:**
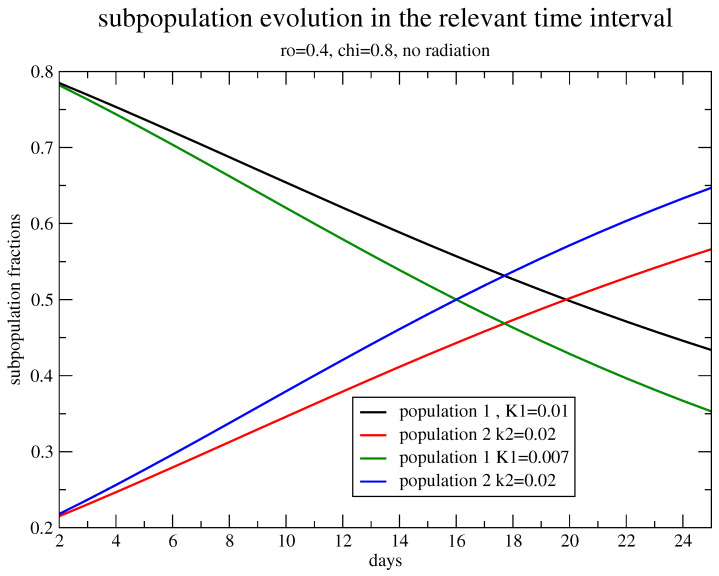
The fraction of the total number of cells of the two strains, growing with different specific rates. Two fractions, 80%/20% of $N_{0}$ and 40%/60% of $N_{\infty }$, with $k_{g}^{\left(1\right)}=0.01,k_{g}^{\left(2\right)}=0.02$ in day ${}^{\left(-1\right)}$, α=0.3 Gy${}^{\left(-1\right)}$, β=0.03 Gy${}^{\left(-2\right)}$. $k_{g}^{\left(1\right)}=0.007$ in day ${}^{\left(-1\right)}$ is shown. The time interval corresponds to the standard treatment of 25 days.

For illustrative purposes, let us show the two population dynamics, with different specific rates for breast cancer in vivo with $N_{\infty }=3.1\times 10^{12}$, $N_{0}=4.8\times 10^{9}$ [6] without any drug for an initial small strain, which, however, gives a large contribution to the carrying capacity $N_{\infty }$. Figure 1 depicts the evolution of the two populations for χ=0.8, ρ=0.4, $k_{g}^{\left(1\right)}=0.01$, $k_{g}^{\left(2\right)}=0.02$ in day ${}^{-1}$ and for $k_{g}^{\left(1\right)}=0.007$ in day ${}^{-1}$.

A more interesting case is the comparison between the survival fraction evaluated for homogeneous and inhomogeneous systems with the same total carrying capacity, initial value $N_{0}$, and uniform irradiation.

Let us consider the homogeneous treatment of 50 Gy with a daily dose of d=2 Gy and assume that the initial total number of cells, $N_{0}$, consists of two fractions, 80%/20% of $N_{0}$, with the parameters $k_{g}^{\left(1\right)}=0.01$ and $k_{g}^{\left(2\right)}=0.02$ in day ${}^{-1}$ and the percentages of the total carrying capacity, $N_{\infty }$, given by 40%/60%. For breast cancer α=0.3 Gy${}^{\left(-1\right)}$, β=0.03 Gy${}^{\left(-2\right)}$.

**Figure 2:**
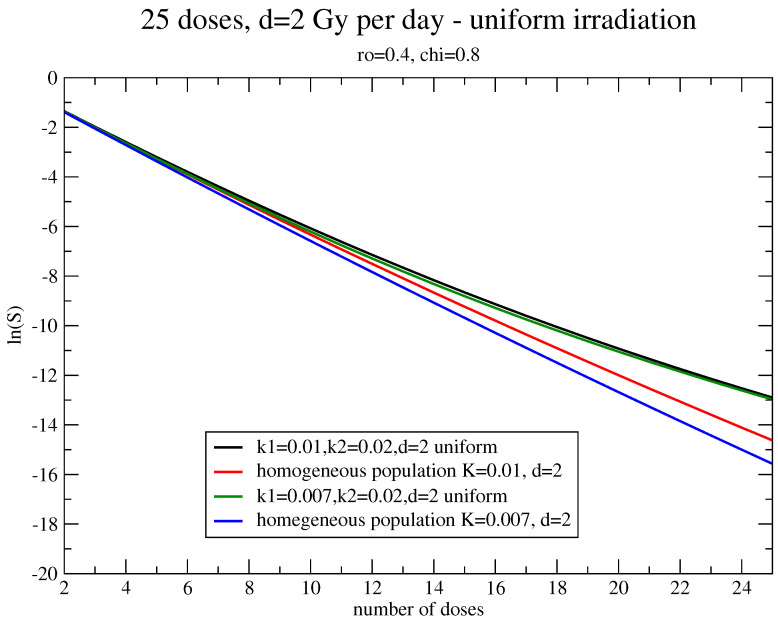
Estimate of the survival fraction with with two fractions, 80%/20% of $N_{0}$ and 40%/60% of $N_{\infty }$, with $k_{g}^{\left(1\right)}=0.01,k_{g}^{\left(2\right)}=0.02$ in day ${}^{\left(-1\right)}$, α=0.3 Gy${}^{\left(-1\right)}$, β=0.03 Gy${}^{\left(-2\right)}$, $k_{g}=k_{g}^{\left(1\right)}$. $k_{g}^{\left(1\right)}=0.007$ in day ${}^{\left(-1\right)}$ is shown.

Figure 2 shows the dependence on the number of doses of the survival fraction $S=N\left(n\right)/N_{0}$. The system consists of a slower and large initial subpopulation (χ=0.8, $k_{g}^{\left(1\right)}=0.01$ in day ${}^{-1}$) and a faster, small strain ($1-\chi =0.2,k_{g}^{\left(2\right)}=0.02$ in day ${}^{-1}$), which gives a substantial contribution to the total carrying capacity (1−ρ=0.6). The results are compared with a homogeneous system with the same values of $N_{0}$, $N_{\infty }$, and $k_{g}=k_{g}^{\left(1\right)}$ in day ${}^{-1}$. In other terms, one evaluates the role of a fast, small strain in the evolution and in the radiotherapy effects of the tumor.

**Figure 3:**
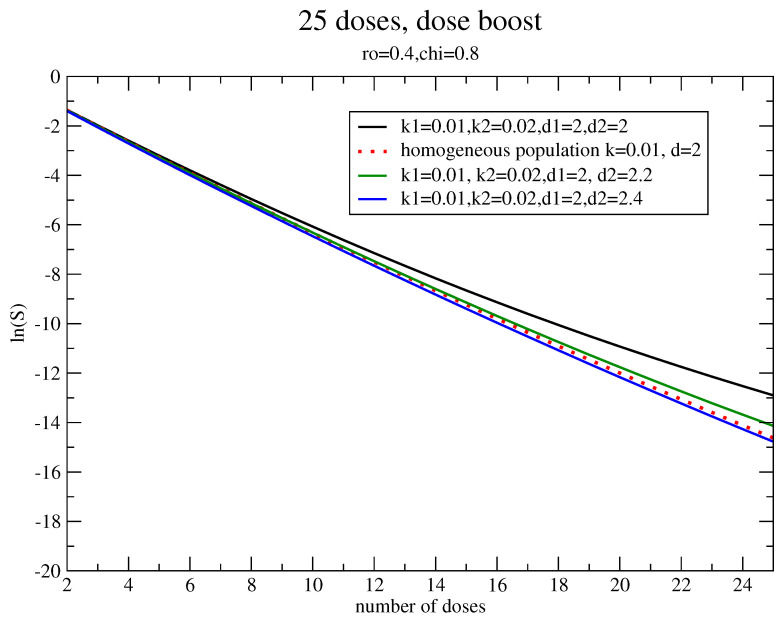
Estimate of the dose-boost with the same parameters of Figure 2.

Therefore, in the case of uniform resistance ($\alpha_{1}=\alpha_{2}$, $\beta_{1}=\beta_{2}$) but different regrowth parameters, the non linearity of the dynamics translates as dose-boosting to have, for the inhomogeneous case, the same survival fraction obtained by uniform irradiation. The increase of the dose for the fast strain is depicted in Figure 3 and turns out to be about 20%.

**Figure 4:**
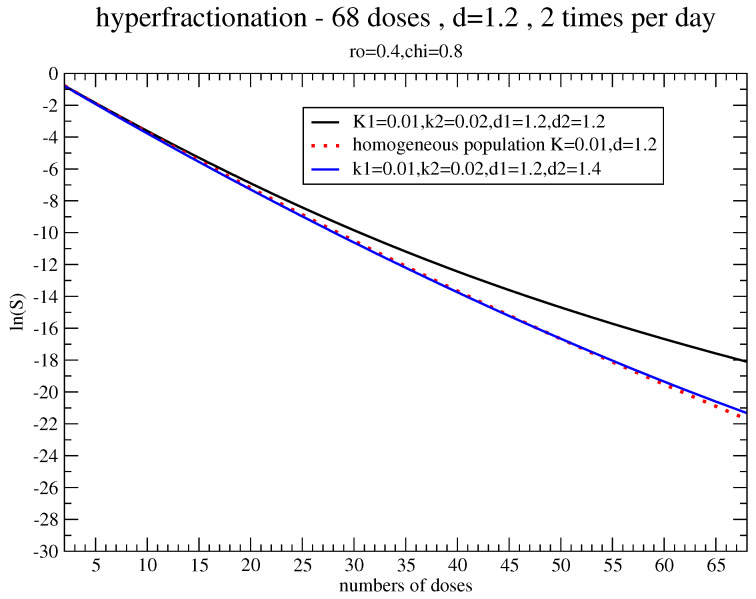
Estimate of the dose-boost with the same parameters of Figure 2 for a hyperfractionated schedule.

**Figure 5:**
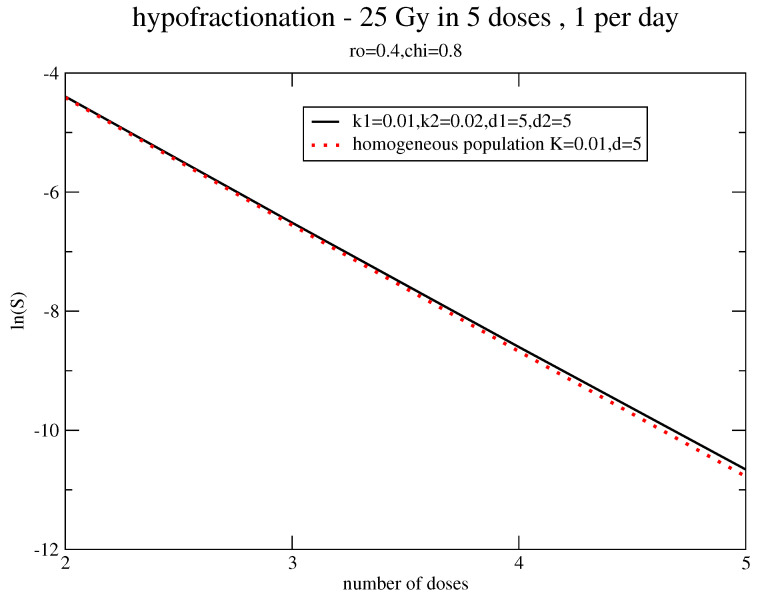
Estimate of the dose-boost with the same parameters of Figure 2 for a hypofractionated schedule.

Since the regrowth effects depend on the interval between the doses, Figure 4 and Figure 5 concern, respectively, the survival probability for the “hyperfractionated” schedule (68 doses, d=1.2 Gy, and two times per day) and the “hypofractionated” one (five doses, 5 Gy, and five days), with the same parameters as in Figure 2, independently of the effective use of these fractionations for breast cancer. In fact, the hyperfractionated treatment requires a dose-boost of about 10% but the hypofractionated schedule completely minimizes the effects of regrowth inhomogeneity.

All previous illustrative analyses considered an equal response of the two subpopulations to radiotherapy, i.e., $\alpha_{1}=\alpha_{2}$ and $\beta_{1}=\beta_{2}$. However, an increased radioresistance of tumor clonogens is directly correlated with unequal specific growth rates due to the various metabolic activities of the cells of the clonal strains. Therefore, for consistency, both features of the subpopulations have to be taken into account. This aspect is discussed in the next subsection for hypoxic strains.

### 3.2. Including Hypoxia

To evaluate the amount of radiation dose to eradicate hypoxic clonal strains, the previous Equations (10)–(12) are now applied for two populations with different parameters of the linear-quadratic model. Let us first consider a small hypoxic component with a low growth rate with respect to a larger subpopulation. The dependence of the oxygen enhancement ratio (OER) on the dose per fraction is included by assuming $\alpha /\alpha_{H}$ = OER and ${\left(\alpha /\beta \right)}_{H}=\left(\alpha /\beta \right)$ OER where $\alpha_{H}$ and $\beta_{H}$ are the hypoxis clonogen subpopulation parameters.

**Figure 6:**
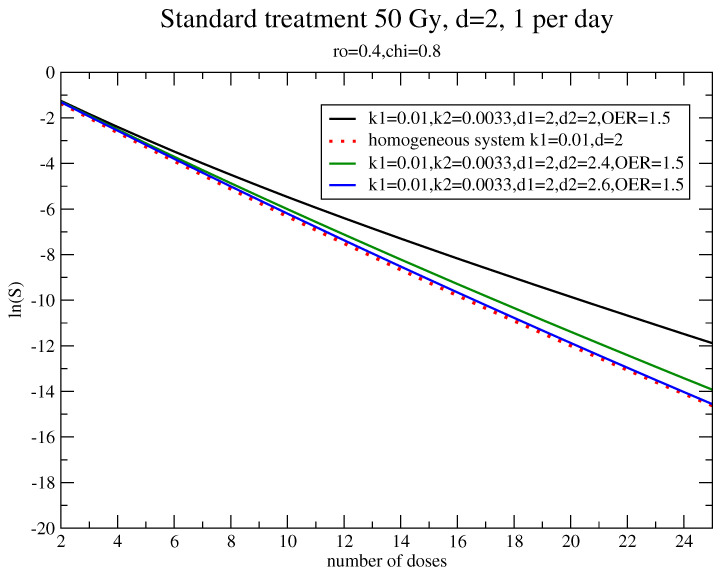
The survival fraction for ρ=0.4, χ=0.8, $k_{g}\left(2\right)=0.0033$, $k_{g}\left(1\right)=k_{g}=0.01$, α=0.3 Gy${}^{\left(-1\right)}$, β=0.03 Gy${}^{\left(-2\right)}$, and OER=1.5.

In Figure 6, the dependence of the survival fraction on the number of doses is depicted for different dose-boosts of the hypoxic component. The initial (pre-treatment) size of the hypoxic component is (1−χ=)20% of the total population. The growth rates are specified by ρ=0.4, χ=0.8, $k_{g}\left(2\right)=0.0033$, $k_{g}\left(1\right)=k_{g}=0.01$, α=0.3 Gy${}^{\left(-1\right)}$, β=0.03 Gy${}^{\left(-2\right)}$, and OER = 1.5. The resulting dose-boost is about 30%.

According to the results in Figure 5, the hypofractionated treatment reduced the effects of the non-linear progression when a small but faster strain was considered. However, for hypoxia, the subpopulation was not only smaller but also slower than the main strain and, therefore, let us evaluate the response to that schedule, including OER.

**Figure 7:**
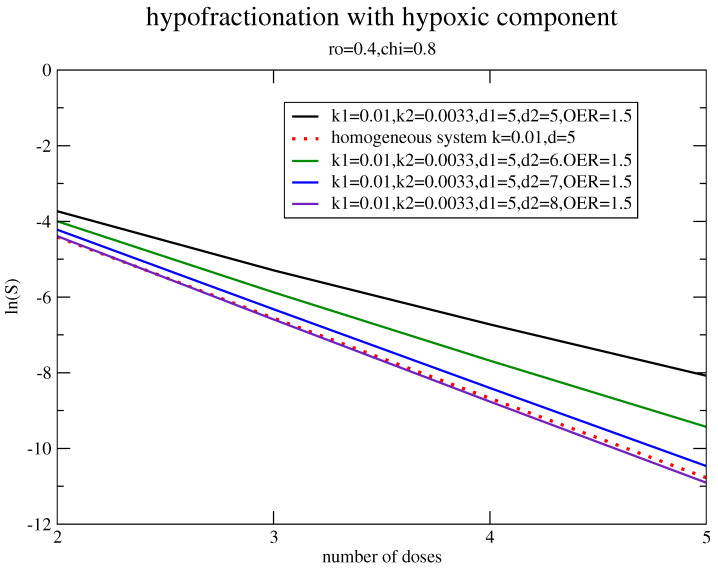
The survival fraction for hypofractionation with ρ=0.4, χ=0.8, $k_{g}\left(2\right)=0.0033$, $k_{g}\left(1\right)=k_{g}=0.01$, α=0.3 Gy${}^{\left(-1\right)}$, β=0.03 Gy${}^{\left(-2\right)}$, and OER=1.5.

The result is reported in Figure 7: a substantial enhancement of the daily dose is required.

## 4. Toward a Patient Oriented Implementation of the Algorithm

The redefinition of the doses when subpopulations with different specific rates and radioresistances contribute to the tumor progression depend on the parameters ρ and χ and on the ratio $k_{g}\left(2\right)/k_{g}\left(1\right)$. Let us discuss the possibility of estimating such parameters with a view toward patient-oriented therapy. For tumor growth, there is a well known linear correlation between the two parameters, $\alpha_{g}$ and $k_{g}$, of the GL, i.e., $\alpha /k_{g}=\gamma$ [5,8]. Accordingly, a single parameter describes the GL progression rate of a specific strain.

To date, PET-CT processing programs allow the following numerical parameters to be obtained: the volume of a region of interest drawn by us (for example of the whole tumor if the 18-F-FDG metabolism tracer is used or of its hypoxic component if the 18-F-FMISO hypoxia tracer is used); the SUV (standardized uptake value) can be the maximum or average and can be calculated with all tracers used; the TLG (total lesion glycolysis) is valid for 18-FDG, which indicates the total amount of metabolism tracer contained in the area of interest that we can draw around the metabolically active lesion; the TBR (tumor to blood ratio) in which we can calculate, for each pixel, the ratio between the F-MISO concentration and the blood concentration obtained from a venous blood sample during the acquisition of the PET image; and the TMR (tumor to muscle ratio), a simpler calculated ratio between the concentration of F-MISO in the hypoxic tumor tissue and that in a reference region containing well-oxygenated tissue.

Using the volume and SUV variations of the areas with FMISO-uptake, one sets the parameter ρ. The other important parameter, χ could be estimated as follows. The ratio of the metabolic rates of the two subpopulations, *g*, estimated by PET, can be considered proportional to the corresponding ratio of the parameters driving the growth rate $\gamma^{1}/\gamma^{2}$. Therefore, (see Equation (4))

(13) $$g=c\frac{\gamma^{1}}{\gamma^{2}}=c\frac{ln[N_{\infty }^{1}/N_{0}^{1}]}{ln[N_{\infty }^{2}/N_{0}^{2}]}=c\frac{ln[N_{\infty }\rho /\chi N_{0}]}{ln[N_{\infty }\left(1-\rho \right)/\left(1-\chi \right)N_{0}]},$$

where *c* is a constant. Therefore, χ is the solution of the equation

(14) $${\left(1-\chi \right)}^{g/c}/\chi ={\left(N_{\infty }/N_{0}\right)}^{\left(g/c-1\right)}\frac{{\left(1-\rho \right)}^{g/c}}{\rho }.$$

Since $N_{\infty }/N_{0}$ is large and 0<χ<1, g/c≃1. However, the precise determination by PET of the relation between the metabolic activities and the specific growth rates is not an easy task and, for a first numerical indication of the dose-boost, one can implement the proposed algorithm by assuming χ≃ρ (i.e., the same weight of the strains at the initial observation and in the carrying capacities) and increasing χ (i.e., decreasing 1−χ) since the second strain has a lower rate and larger radioresistance.

In summary, the undetermined parameters are $k_{g}$ and χ, since the other ones in Equations (10)–(12), are fixed. The distribution function of $k_{g}$ for many cancer phenotypes is known [6,8] and the variation of χ gives a band of values for the dose-boost, for different OER values.

## 5. Conclusions

The results of the previous sections suggest how to apply the proposed method to evaluate the modification of irradiation treatment when the cancer progression originates from two (or more) cell groups with different specific replication rates and radioresistances. An analogous analysis was carried out for chemotherapy, implying modification of the Simon–Norton hypothesis [22].

Cellular inhomogeneity, partially promoted by a non-uniform oxygen supply, produces different rates of cell proliferation inside tumors, which could negatively affect the responsiveness to oncologic treatments. Such an intra-tumoral dose diversification preludes personalized radiotherapy with new technological facilities that are able to identify different metabolic areas within tumor tissues. Although PET CT is not suitable to differentiate various subclones during RT in everyday practice, some hypoxic specific tracers are still available, (i.e., FAZA and F-MISO) and are able to depict a baseline oxygen tissue map [23,24].

The proposed approach involves two-population dynamics; however, tumoral oxygenation can change quantitatively, both spatially and temporally. In fact, the above simplification has to be generalized for a more complete description of the tumor response to radiation by a larger number of subpopulations to consider some other fundamental parameters, such as the tumor stroma and microenvironment, cell signaling, and immune cascades.

## Figures and Tables

**Figure A1:**
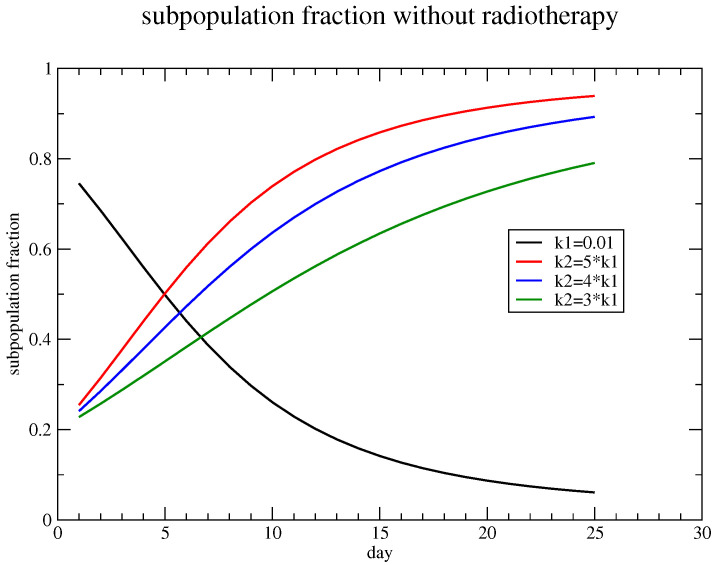
Subpopulation fractions for different rates of the fast/slow subpopulation, with the same parameters as in Figure 1.

## Data Availability

Not applicable.

## Appendix A

Let us call $N_{0}$ the initial cell number consisting of $N_{0}^{\left(1\right)}=\chi N_{0}$ and $N_{0}^{\left(2\right)}=\left(1-\chi \right)N_{0}$. The firts dose, at time t=0 (the possible delay between the intial clinical observation and first dose can be easily included) gives

(A1) $$N_{1}^{\left(1\right)}=N_{0}^{\left(1\right)}e^{\left[-\alpha f\left(1\right)d-\beta f{\left(1\right)}^{2}d^{2}\right]},$$

(A2) $$N_{1}^{\left(2\right)}=N_{0}^{\left(2\right)}e^{\left[-\alpha \left(1-f\left(1\right)\right)d-\beta {\left(1-f\left(1\right)\right)}^{2}d^{2}\right]},$$

with $N_{1}=N_{1}^{\left(1\right)}+N_{1}^{\left(2\right)}$.

By defining the fraction of the carrying capacity due to strain 1 and strain 2, respectively, as $N_{\infty }^{\left(1\right)}=\rho N_{\infty }$ and $N_{\infty }^{\left(2\right)}=\left(1-\rho \right)N_{\infty }$, the evolution after the first dose and before the second one, $1^{+}\to 2^{-}$, according to GL gives

(A3) $$N_{2^{-}}^{\left(1\right)}=N_{0}^{\left(1\right)}e^{\lambda_{1}ln\left(\frac{\rho N_{\infty }}{\chi N_{0}}\right)+\left[-\alpha d-\beta d^{2}\right]\left(1-\lambda_{1}\right)}$$

(A4) $$N_{2^{-}}^{\left(2\right)}=N_{0}^{\left(2\right)}e^{\lambda_{2}ln\left(\frac{\left(1-\rho \right)N_{\infty }}{\left(1-\chi \right)N_{0}}\right)+\left[-\alpha d-\beta d^{2}\right]\left(1-\lambda_{2}\right)}$$

where $N_{2^{-}}=N_{2^{-}}^{\left(1\right)}+N_{2^{-}}^{\left(2\right)}$,

(A5) $$\lambda_{1}=1-e^{-k_{1}\Delta t}$$

and

(A6) $$\lambda_{2}=1-e^{-k_{2}\Delta t},$$

with $k_{1},k_{2}$ as the GL parameters. By iterative procedure,

(A7) $$N{\left(n\right)}^{\left(1\right)}=N_{0}^{\left(1\right)}e^{ln\left(N_{\infty }^{\left(1\right)}/N_{0}^{\left(1\right)}\right)I_{1}\left(n\right)-\left(\alpha d+\beta d^{2}\right)L_{1}\left(n\right)}$$

where

(A8) $$I_{1}\left(n\right)=1-e^{-\left(n-1\right)k_{g}^{\left(1\right)}\Delta t}$$

and

(A9) $$L_{1}\left(n\right)=\frac{1-e^{-nk_{g}^{\left(1\right)}\Delta t}}{1-e^{-k_{g}^{\left(1\right)}\Delta t}},$$

with analogous formulas for the second cell group by the substitutions $\rho \to \left(1-\rho \right),\chi \to \left(1-\chi \right),and\;\lambda_{1}\to \lambda_{2}$.

If the two strains also have different resistances to radiotherapy, the previous equations can be easily generalized by considering $\alpha \to \alpha_{1}$ or $\alpha_{2}$ and $\alpha \to \beta_{1}$ or $\beta_{2}$. The results in Section 3 are based on $N\left(n\right)=N{\left(n\right)}^{\left(1\right)}+N{\left(n\right)}^{\left(2\right)}$ and on Equations (21)–(23) and the analogous equations for strain 2.

For sake of completeness, in Figure A1 we show the growth pattern of the two subpopulations without radiotherapy (i.e., α=β=0) for different ratios of the growth parameter $k_{1},k_{2}$, with the same parameters of Figure 1.
